# Association between aspartate aminotransferase to alanine aminotransferase ratio and the risk of diabetes in Chinese prediabetic population: A retrospective cohort study

**DOI:** 10.3389/fpubh.2022.1045141

**Published:** 2023-01-04

**Authors:** Xiaoqing Wang, He Li, Lin Ji, Jing Cang, Hang Zhao

**Affiliations:** ^1^Department of Anaesthesiology, Zhongshan Hospital, Fudan University, Shanghai, China; ^2^Department of Anesthesiology, Affiliated Shuguang Hospital, Shanghai University of Traditional Chinese Medicine, Shanghai, China; ^3^Department of Anesthesiology, Yancheng Third People's Hospital, The Yancheng School of Clinical Medicine of Nanjing Medical University, Yancheng, Jiangsu, China

**Keywords:** aspartate aminotransferase, alanine aminotransferase, prediabetes, association, Kaplan-Meier curve, subgroup analysis

## Abstract

**Background:**

Accumulating evidence has revealed that the aspartate aminotransferase to alanine aminotransferase (AST/ALT) ratio is a promising novel biomarker for insulin resistance (IR) and metabolic diseases. However, research on the association between the AST/ALT ratio and the incidence of diabetes progressing from prediabetes remains lacking. Herein, this study aimed to evaluate the relationship between the baseline AST/ALT ratio and risks of diabetes in patients with prediabetes.

**Methods:**

This was a retrospective cohort study involving a total of 82,683 participants across 32 regions and 11 cities in China from 2010 to 2016. Data was obtained based on the DATADRYAD database from the health check screening program. Participants were stratified according to the interquartile range of the AST/ALT ratio (groups Q1 to Q4). The Cox proportional hazard model and smooth curve fitting were used to explore the relationship between the baseline AST/ALT ratio and the risk of diabetes in prediabetic patients. In addition, subgroup analysis was used to further validate the stability of the results.

**Results:**

The mean age of the selected participants was 49.9 ± 14.0 years, with 66.8% of them being male. During the follow-up period 1,273 participants (11.3%) developed diabetes progressing from prediabetes during the follow-up period. Participants who developed diabetes were older and were more likely to be male. The fully-adjusted Cox proportional hazard model revealed that the AST/ALT ratio was negatively associated with the risk of diabetes in prediabetic patients (HR = 0.40, 95% CI: 0.33 to 0.48, *P* < 0.001). Higher AST/ALT ratio groups (Q4) also presented with a lower risk of progressing into diabetes (HR = 0.35, 95% CI: 0.29 to 0.43, *P* < 0.001, respectively) compared with the lowest quintile group (Q1). Through subgroup analysis and interaction tests, it was found that the association stably existed in all subgroup variables, and there were a stronger interactive effects in people with age < 45 years, and TG ≤ 1.7 mmol/L in the association between AST/ALT ratio and diabetes incidences in patients with prediabetes (*P* for interaction < 0.05).

**Conclusion:**

According to our study, a higher AST/ALT ratio is associated with a lower risk of progressing into diabetes from prediabetes. Regular monitoring of AST/ALT ratio dynamics and corresponding interventions can help prevent or slow prediabetes progression for diabetes.

## Introduction

Diabetes is emerging as one of the most important public health challenges of the 21st century. The World Health Organization (WHO) documents that diabetes caused an estimated 1.6 million deaths in 2016 and was the seventh leading cause of death globally (1). By the end of 2015, the global number of people with diabetes had reached 415 million. And this number is predicted to increase to 642 million by 2040 (2, 3). The prevalence of diabetes among adults in China has been reported to be as high as 12.8%, with the total number of patients in mainland China estimated at 129.8 million, which ranks first in the world. By 2,035, the number of diabetes cases in China is expected to reach 143 million (4, 5). Diabetes is usually accompanied by severe complications, including retinopathy and blindness, renal failure, heart failure, coronary artery disease, stroke, as well as peripheral neuropathy (6, 7). In 2015, the total cost for treating diabetes and its associated complications was USD 673 billion, which is anticipated to increase to USD 802 billion by the year 2040 (8). Diabetes and its complications have a detrimental impact on patients, families, and society, and impose a significant economic burden. Thus, early diagnosis and intervention are important, especially for prediabetes, to reduce the serious harm caused by diabetes and its complications.

Prediabetes is defined as blood glucose concentrations above normal but below the threshold for diabetes. It includes impaired fasting glucose (IFG) and impaired glucose tolerance (IGT). Recent studies indicate that more than one-third of individuals have prediabetes in China. It is estimated that up to 70% of people with pre-diabetes will develop diabetes over many years (9, 10). Most people with prediabetes do not have any obvious clinical symptom, which is usually ignored by people. In fact, prediabetes is a high-risk metabolic state that predicts an increased probability of developing diabetes and may itself be associated with health risks and complications (9). Recent studies have shown that prediabetes is associated with an increased risk of all-cause mortality and cardiovascular disease, such as atherosclerotic cardiovascular disease, heart failure, etc (11–13). However, currently, there are no specific and feasible methods for diabetes or prediabetes prediction. Therefore, there is an urgent global need for simple, sensitive, and cost-effective screening strategies to enhance the early identification and prevention of diabetes or prediabetes.

The liver is a key organ of systemic metabolism and contributes significantly to the development of insulin resistance and type 2 diabetes mellitus (T2DM) (14–16). Non-alcoholic fatty liver disease (NAFLD) has a bidirectional association with T2DM. Patients with NAFLD usually have IR. Meanwhile, many T2DM patients develop NAFLD with the inflammatory complication, nonalcoholic steatohepatitis (NASH) (16). The major serum liver enzymes (AST and ALT) are the most sensitive indicators for the clinical evaluation of liver cell damage and death (17). A well-characterized multiethnic cohort trial named the Insulin Resistance Atherosclerosis Study (IRAS) found that liver injury markers, including AST and ALT, were closely associated with T2DM risk (18). The serum AST/ALT ratio concept was first proposed by De Ritis and is known as the De Ritis Ratio. The De Ritis ratio is used to diagnose various chronic liver diseases, including alcoholic and non-alcoholic fatty liver diseases (NAFLD), hepatitis, and autoimmune liver diseases (19–22). Besides, this ratio is also associated with other non-liver diseases, such as metabolic syndromes (MS), T2DM, cardiovascular diseases, acute stroke, and several malignant tumors (23–28). Several studies have investigated the relationship between the indicators of liver function (AST, ALT, and AST/ALT) and the risk of T2DM (29–33). However, studies of the relationship between the AST/ALT ratio and the risk of diabetes in pre-diabetic patients are very limited currently. Therefore, the purpose of this study was to clarify the association between the AST/ALT ratio and the risk of progression to diabetes from prediabetes in Chinese adults.

## Methods

### Data sources

We obtained the using data from the Dryad Digital Repository (www.Datadryad.org). The Dryad Digital Repository is a Data Platform aiming to make published scientific articles shareable, freely available for secondary use, and citable. We downloaded the raw data free of charge. In the present study, we cited the Dryad data package (Data from: Association of body mass index and age with incident diabetes in Chinese adults: a population-based cohort study. Dryad Digital Repository. https://doi.org/10.1136/bmjopen-2018-021768) (34). Since Chen et.al have authorized the ownership of the original data to the datadryad website. According to Dryad's Terms of Service, we can use this data to perform secondary data analysis on a different hypothesis without infringing on the authors, rights. The initial study conducted by Chen et.al was approved by the Rich Healthcare Group review committee. As this study was retrospective, no ethical approval was required for this secondary analysis. This study followed the principles of the Declaration of Helsinki. All methods were carried out in accordance with the relevant guidelines and regulations, including the statements in the declarations section. All reporting followed the Strengthening the Reporting of Observational Studies in Epidemiology (STROBE) guidelines (35).

### Study participants

**Figure 1:**
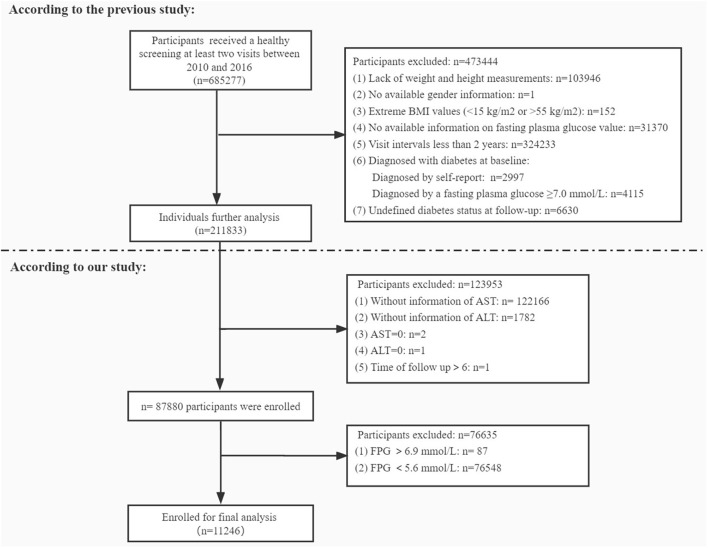
Flow chart of the study population.

In the present study, we retrospectively analyzed data of 6,85,277 adult Chinese participants aged 20 years and over, who underwent a healthy screening at least two visits between 2010 and 2016 across 32 sites and 11 cities in China (Beijing, Shanghai, Guangzhou, Shenzhen, Nanjing, Wuhan, Hefei, Chengdu, Suzhou, Changzhou, Nantong). These clinical records were extracted from a computerized database established by the Rich Healthcare Group in China. This study was approved by the Rich Healthcare Group Review Board before data collection. After initial screening 6,74,031 participants were excluded due to different reasons presented in Figure 1. Consequently 11,246 participants were finally included to assess the relationship between the AST/ALT ratio and incidence of diabetes in the prediabetic population.

### Study design and measurement of covariant

As mentioned in the previous research, participants were asked to finish a questionnaire concerning general demographic (age, gender), living habits (smoking status, drinking status), personal health and medication history, and family history of chronic disease (family history of diabetes) in as much detail as possible at each healthy examination. Height, weight, and blood pressure were measured by trained staff. BMI was obtained by dividing weight (kg) by the square of height (m). Blood pressure was measured with a standard mercury sphygmomanometer. Drinking status is divided into current drinker, ever drinker, and never drinker. Smoking status is divided into current smoker, ever smoker, and never smoker. For all participants, fasting venous blood samples were collected after at least 10 h of fasting for each examination. Serum triglyceride (TG), total cholesterol (TC), low-density lipoprotein cholesterol (LDL-C), high-density lipoprotein cholesterol (HDL-C), AST, and ALT were measured using autoanalyzer (Beckman 5800). Determination of serum glucose levels using the glucose oxidase method on an automated analyser (Beckman 5800). The target independent variable is AST/ALT ratio obtained at baseline and recorded as a continuous variable. The dependent variable is the incidence of diabetes progressing from prediabetes (dichotomous variable).

### Definitions

The AST/ALT ratio was defined as AST divided by ALT. The diagnostic criteria of diabetes were described as follows: fasting plasma glucose > 7.00 mmol/L and/or self-reported diabetes during the follow-up period (34). According to the American Diabetes Association 2022 criteria, prediabetes is defined as participants who had an FPG level between 5.6 and 6.9 mmol/L (36).

### Statistical analysis

In order to investigate whether the AST/ALT ratio of the selected participants in the prediabetic stage is related to the incidence of diabetes, the procedure of statistical analysis includes 6 main steps. First, for missing values, we used multiple imputations to replace based on 5 replications. Second, the baseline characteristics of participants were described according to the quartile of AST/ALT ratio and with/ without diabetes. Continuous variables were presented using the mean and the standard deviation (SD) when normally distributed or otherwise the median and interquartile range (IQR). Categorical variables were presented as proportions and percentages of the total. Comparisons between groups were assessed using the X2 test or Fisher's exact test (for categorical variables) and Student *t*-test (for continuous variables) or Mann-Whitney U-test (for continuous variables). Third, univariate and multivariate cox proportional hazards models were built to evaluate HR with a 95% confidence interval (CI) of diabetes for AST/ALT ratio. Fourth, we used the cox proportional hazards model to calculate the association between AST/ALT ratio and the incident diabetes with baseline AST/ALT ratio fitted as continuous (per SD increment) or categorical (tertiles) variables. Model I did not adjust any confounders. Model II adjusted for age and gender. Model III additionally adjusted for age, gender, BMI, SBP, DBP, TG, TC, HDL-C, LDL-C, BUN, Scr, smoking status, drinking status, and family history of diabetes. Fifth, the restricted cubic spline model was used for the dose-response analysis. Sixth, based on stratified cox proportional hazard models, we assessed the consistency of the association between the AST/ALT ratio and the incidence of diabetes by subgroup analyses. We transformed continuous variables into categorical variables regarding clinical cut points or using quartile, and then tested for interaction. Finally, the Kaplan–Meier probabilities of diabetes-free survival were compared using the log-rank test among the quartile of AST/ALT ratio. All the analyses were performed with R statistics software and FreeStatistics software. A two-tailed test was performed and *P* < 0.05 was considered statistically significant.

## Results

### Baseline characteristics of selected participants

A total of 11,246 participants were selected for the final data analysis (Figure 1). The mean age of the selected participants was 49.9 ± 14.0 years, of which approximately 66.8% were male. The average follow-up year was 3.0 ± 0.9 years, and 1,273 participants (11.3%) developed diabetes progressing from prediabetes during the follow-up period. The mean value of the AST/ALT ratio was 1.2 ± 0.5, and the mean value of BMI was 24.8 ± 3.3 kg/m2. The baseline characteristics of participants based on quartiles of AST/ALT ratio and the proportion of diabetes occurring were presented in Tables 1, 2, respectively. Among quartiles of the AST/ALT ratio, there were great differences in all baseline characteristics. Participants with the highest quartiles of the AST/ALT ratio had the lowest BMI, SBP, DBP, TC, TG, LDL-C, BUN, and Scr (*P* < 0.05; Table 1). In addition, participants in the group with the lowest AST/ALT ratio had the lowest age and HDL-C values (*P* < 0.05; Table 1). When compared with participants without diabetes during follow-up, participants who developed diabetes were older, more likely to be male, had greater values of BMI, SBP, DBP, TC, TG, had lower levels of HDL-C, AST/ALT ratio, had a higher proportion of current smoker and drinker, and more likely to have the family history of diabetes (*P* < 0.05; Table 2).

**Table 1:** Baseline characteristics of participants stratified by quartiles of AST/ALT ratio.

**Variables**	**AST/ALT ratio quartile**				***P*-value**
	**Q1 (< 0.8155)**	**Q2 (0.8155–1.0830)**	**Q3 (1.0830–1.4149)**	**Q4 (>1.4149)**	
Participants	2,809	2,800	2,825	2,812	
Age (years)	44.3 ± 11.2	49.8 ± 12.9	52.3 ± 14.2	53.4 ± 15.6	< 0.001
Gender, *n (%)*					< 0.001
Male	2,431 (86.5)	2,075 (74.1)	1,719 (60.8)	1,291 (45.9)	
Female	378 (13.5)	725 (25.9)	1,106 (39.2)	1,521 (54.1)	
BMI (Kg/m2)	26.5 ± 3.2	25.3 ± 3.1	24.3 ± 3.1	23.2 ± 3.1	< 0.001
SBP (mmHg)	128.4 ± 16.0	127.6 ± 17.3	127.8 ± 18.5	126.6 ± 18.9	0.002
DBP (mmHg)	80.4 ± 10.7	78.9 ± 10.9	78.1 ± 11.3	76.4 ± 11.4	< 0.001
TG (mmol/L)	1.8 (1.2, 2.7)	1.6 (1.1, 2.3)	1.3 (0.9, 1.9)	1.1 (0.8, 1.5)	< 0.001
TC (mmol/L)	5.1 ± 1.0	5.0 ± 0.9	5.0 ± 0.9	4.9 ± 0.9	< 0.001
HDL-C (mmol/L)	1.3 ± 0.3	1.3 ± 0.4	1.4 ± 0.3	1.4 ± 0.3	< 0.001
LDL (mmol/L)	3.0 ± 0.7	2.9 ± 0.7	2.9 ± 0.7	2.8 ± 0.7	< 0.001
AST (mmol/L)	31.3 ± 14.8	25.6 ± 9.9	24.2 ± 8.6	24.2 ± 10.5	< 0.001
ALT (mmol/L)	51.0 ± 31.1	27.3 ± 11.2	19.7 ± 7.3	13.8 ± 5.9	< 0.001
AST/ALT	0.7 ± 0.1	0.9 ± 0.1	1.2 ± 0.1	1.8 ± 0.5	< 0.001
BUN (mmol/L)	5.1 ± 1.2	5.1 ± 1.2	5.0 ± 1.3	5.0 ± 1.3	0.013
Scr (mmol/L)	77.0 ± 14.0	75.3 ± 15.7	73.1 ± 16.2	70.7 ± 17.5	< 0.001
Smoking status, *n (%)*					< 0.001
Current smoker	806 (28.7)	657 (23.5)	531 (18.8)	386 (13.7)	
Ever smoker	176 (6.3)	131 (4.7)	108 (3.8)	74 (2.6)	
Never smoker	1,827 (65)	2,012 (71.9)	2,186 (77.4)	2,352 (83.6)	
Drinking status, *n (%)*					< 0.001
Current drinker	116 (4.1)	117 (4.2)	129 (4.6)	137 (4.9)	
Ever drinker	701 (25)	572 (20.4)	476 (16.8)	313 (11.1)	
Never drinker	1,992 (70.9)	2,111 (75.4)	2,220 (78.6)	2,362 (84)	
Family history of diabetes, *n (%)*					0.007
No	2,720 (96.8)	2,735 (97.7)	2,775 (98.2)	2,748 (97.7)	
Yes	89 (3.2)	65 (2.3)	50 (1.8)	64 (2.3)	
Follow-up (years)	3.0 ± 0.9	3.0 ± 0.9	3.0 ± 0.9	3.0 ± 0.9	< 0.001
Incident diabetes, *n (%)*					< 0.001
No	2,366 (84.2)	2,434 (86.9)	2,537 (89.8)	2,636 (93.7)	
Yes	443 (15.8)	366 (13.1)	288 (10.2)	176 (6.3)	

Data are shown as mean ± standard deviation or number (%).

AST, aspartate aminotransferase; ALT, alanine aminotransferase; FPG, fasting plasma glucose; DM, diabetes mellitus; BMI, body mass index; SBP, systolic blood pressure; DBP, diastolic blood pressure; TG, triglyceride; Scr, serum creatinine; BUN, blood urea nitrogen; TC, total cholesterol; HDL-C, high-density lipid cholesterol; LDL-C, low-density lipid cholesterol.

**Table 2:** Baseline characteristics of study participants with/without diabetes.

**Variables**	**Total (*n* = 11246)**	**Subgroups of patients**		***P*-value**
		**Non-diabetes** **(*****n*** = **9973)**	**diabetes** **(*****n*** = **1273)**	
Age (years)	49.9 ± 14.0	49.2 ± 14.1	55.6 ± 12.3	< 0.001
Gender, *n* (%)				< 0.001
Male	7,516 (66.8)	6,608 (66.3)	908 (71.3)	
Female	3,730 (33.2)	3,365 (33.7)	365 (28.7)	
BMI (Kg/m2)	24.8 ± 3.3	24.7 ± 3.3	26.2 ± 3.3	< 0.001
SBP (mmHg)	127.6 ± 17.7	126.9 ± 17.5	132.7 ± 18.6	< 0.001
DBP (mmHg)	78.4 ± 11.2	78.1 ± 11.1	81.0 ± 11.6	< 0.001
TG (mmol/L)	1.4 (1.0, 2.1)	1.4 (0.9, 2.1)	1.7 (1.2, 2.6)	< 0.001
TC (mmol/L)	5.0 ± 0.9	5.0 ± 0.9	5.1 ± 1.0	< 0.001
HDL-C (mmol/L)	1.3 ± 0.3	1.3 ± 0.3	1.3 ± 0.4	0.037
LDL (mmol/L)	2.9 ± 0.7	2.9 ± 0.7	2.9 ± 0.7	0.632
ALT (mmol/L)	27.9 ± 22.3	27.0 ± 21.1	35.3 ± 28.6	< 0.001
AST/ALT (mmol/L)	1.2 ± 0.5	1.2 ± 0.5	1.0 ± 0.4	< 0.001
AST (mmol/L)	26.3 ± 11.6	26.0 ± 11.2	29.3 ± 14.0	< 0.001
BUN (mmol/L)	5.0 ± 1.3	5.0 ± 1.3	5.1 ± 1.3	0.328
Scr (umol/L)	74.0 ± 16.1	74.0 ± 16.0	74.1 ± 17.2	0.861
Smoking status, *n* (%)				< 0.001
Current smoker	2,380 (21.2)	2,027 (20.3)	353 (27.7)	
Ever smoker	489 (4.3)	402 (4)	87 (6.8)	
Never smoker	8,377 (74.5)	7,544 (75.6)	833 (65.4)	
Drinking status, *n* (%)				< 0.001
Current drinker	499 (4.4)	412 (4.1)	87 (6.8)	
Ever drinker	2,062 (18.3)	1,811 (18.2)	251 (19.7)	
Never drinker	8,685 (77.2)	7,750 (77.7)	935 (73.4)	
Family history of diabetes, *n* (%)				0.001
No	10,978 (97.6)	9,752 (97.8)	1,226 (96.3)	
Yes	268 (2.4)	221 (2.2)	47 (3.7)	
Follow-up (years)	3.0 ± 0.9	3.0 ± 0.9	3.3 ± 0.9	< 0.001

BMI, body mass index; SBP, systolic blood pressure; DBP, diastolic blood pressure; TG, triglyceride; AST, aspartate aminotransferase; ALT, alanine aminotransferase; FPG, fasting plasma glucose; TC, total cholesterol; HDL-C, high-density lipid cholesterol; Scr, serum creatinine; BUN, blood urea nitrogen; LDL-C, low-density lipid cholesterol.

### Risk factors for diabetes in the prediabetic population

As shown in Table 3. By univariate analysis, we found that age, BMI, SBP, DBP, TG, HDL-C, AST/ALT, AST, ALT, family history of diabetes, smoking, and drinking status were correlated with the incidence of diabetes in prediabetic patients (all *P* < 0.05). Furthermore, after adjusting for potential confounding factors according to univariate analysis, the multivariate analysis revealed that age, BMI, DBP, TG, HDL-C, AST/ALT, AST, ALT, and family history of diabetes have a significant association with the incident of diabetes progressing from prediabetes.

**Table 3:** Results of univariate and multivariate analysis and risk factors of diabetes.

**Covariables**	**Univariate analysis**		**Multivariate analysis**	
	**HR (95%CI)**	***P*** **-value**	**HR (95%CI)**	***P*** **-value**
Age (years)	1.03 (1.02,1.03)	< 0.001	1.02 (1.01–1.02)	< 0.001
**Gender**				
Male	Ref			
Female	0.89 (0.79,1.01)	0.063		
BMI (Kg/m2)	1.11 (1.09,1.12)	< 0.001	1.07 (1.05–1.09)	< 0.001
SBP (mmHg)	1.02 (1.01,1.02)	< 0.001	1.00 (1.00–1.01)	0.097
DBP (mmHg)	1.02 (1.01,1.02)	< 0.001	1.01 (1.00–1.01)	0.027
TC (mmol/L)	1.04 (0.98,1.10)	0.215		
TG (mmol/L)	1.12 (1.09,1.15)	< 0.001	1.09 (1.06–1.12)	< 0.001
HDL-C (mmol/L)	1.55 (1.39,1.73)	< 0.001	1.64 (1.5–1.79)	< 0.001
LDL (mmol/L)	1.00 (0.93,1.08)	0.933		
ALT (mmol/L)	1.01 (1.01,1.01)	< 0.001	1.01 (1.00–1.01)	< 0.001
AST/ALT	0.49 (0.42,0.56)	< 0.001	0.42 (0.35–0.5)	< 0.001
AST (mmol/L)	1.01 (1.01,1.01)	< 0.001	1.01 (1.00–1.01)	< 0.001
BUN (mmol/L)	1.04 (0.99,1.08)	0.106		
Scr (mmol/L)	1.00 (1.00,1.00)	0.294		
**Smoking status**				
Current smoker	Ref		Ref	
Ever smoker	1.34 (1.06,1.70)	0.014	1.66 (1.31–2.1)	< 0.001
Never smoker	0.81 (0.71,0.92)	< 0.001	0.94 (0.83–1.07)	0.327
**Drinking status**				
Current drinker	Ref		Ref	
Ever drinker	0.65 (0.51,0.82)	< 0.001	0.82 (0.64–1.05)	0.111
Never drinker	0.67 (0.54,0.84)	< 0.001	0.83 (0.66–1.03)	0.092
**Family history of diabetes**				
No	Ref		Ref	
Yes	1.59 (1.19,2.12)	0.002	1.76 (1.31–2.36)	< 0.001

BMI, body mass index; SBP, systolic blood pressure; DBP, diastolic blood pressure; TG, triglyceride; AST, aspartate aminotransferase; ALT, alanine aminotransferase; FPG, fasting plasma glucose; TC, total cholesterol; HDL-C, high-density lipid cholesterol; Scr, serum creatinine; BUN, blood urea nitrogen; LDL-C, low-density lipid cholesterol.

**Figure 2:**
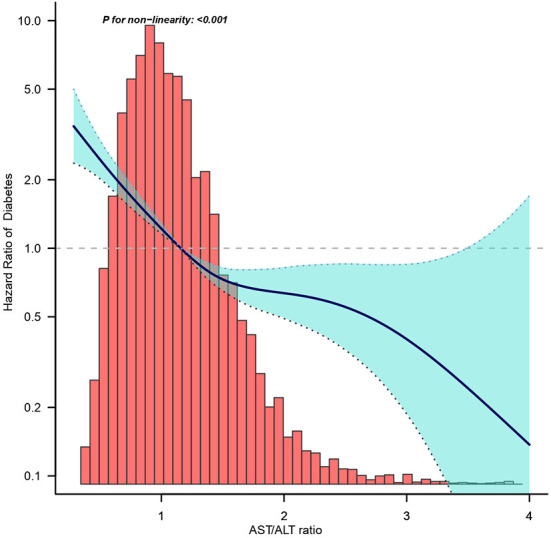
Dose-response relationship between AST/ALT ratio and the incidence of diabetes in prediabetic patients.

Figure 2 shows the Kaplan–Meier curve of the cumulative hazards of incident diabetes risk stratified by AST/ALT ratio categories. The risk of incident diabetes was significantly different between the three AST/ALT groups (Log-rank test, *P* < 0.0001). With an increased AST/ALT ratio, the cumulative risk of incident diabetes gradually decreased, rendering the minimum AST/ALT ratio group with the maximum risk of incident diabetes in prediabetic patients.

### Effect of AST/ALT ratio on the incident of diabetes progressing from prediabetes

In this study, we constructed three models to analyze the independent effects of the AST/ALT ratio on the incidence of diabetes (univariate and multivariate Cox proportional hazard model). The effect sizes [Hazard ratio (HR)] and 95% confidence intervals (CI) were listed in Table 4. When AST/ALT ratio was a continuous variable, AST/ALT ratio showed a negative correlation with the incidence of diabetes progressing from prediabetes in the unadjusted model (model I), for every 1 unit increase in the AST/ALT ratio, the risk of incident of diabetes in the prediabetic patients decreased by 51% (HR = 0.49, 95% CI: 0.42 to 0.56, *P* < 0.001). In the minimum-adjusted model (model II) adjusted for age and gender, the trend did not have obvious changes, the risk of incident diabetes decreased by 69% as the AST/ALT ratio increased (HR = 0.31, 95% CI: 0.26 to 0.37, *P* < 0.001). In the fully-adjusted model (model III) adjusted for age, gender, BMI, SBP, TG, TC, HDL-C, LDL-C, BUN, Scr, smoking status, drinking status, family history of diabetes age, for every 1 unit increase in the AST/ALT ratio, the risk of incident of diabetes decreased by 60% (HR = 0.40, 95% CI: 0.33 to 0.48, *P* < 0.001). For the purpose of sensitivity analysis, we converted the AST/ALT ratio from the continuous variable to the categorical variable (quartiles of AST/ALT ratio). When comparing with the lowest quartile of AST/ALT ratio, the multivariate HRs for the incident of diabetes were 0.71 (0.62–0.82) for Q2, 0.50 (0.42–0.59) for Q3, 0.35 (0.29–0.43) for Q4, The *P* for trend of AST/ALT ratio with categorical variables in the fully-adjusted model was consistent with the result when AST/ALT ratio was a continuous variable.

**Table 4:** Relationship between AST/ALT ratio and the risk of diabetes in prediabetic patients in different models.

**Exposure**	**Model I** **(HR, 95%CI)**	***P*-value**	**Model II** **(HR, 95%CI)**	***P*-value**	**Model III** **(HR, 95%CI)**	***P*-value**
AST/ALT ratio	0.49 (0.42–0.56)	< 0.001	0.31 (0.26–0.37)	< 0.001	0.40 (0.33–0.48)	< 0.001
**AST/ALT ratio quartile**						
Q1	Reference		Reference		Reference	
Q2	0.80 (0.69–0.92)	0.001	0.63 (0.54–0.72)	< 0.001	0.71 (0.62–0.82)	< 0.001
Q3	0.59 (0.51–0.68)	< 0.001	0.41 (0.35–0.49)	< 0.001	0.50 (0.42–0.59)	< 0.001
Q4	0.43 (0.36–0.51)	< 0.001	0.27 (0.22–0.33)	< 0.001	0.35 (0.29–0.43)	< 0.001
*P* for Trend	< 0.001		< 0.001		< 0.001	

Results are shown as hazard ratios (HRs) with 95% confidence intervals (CIs). AST, aspartate aminotransferase; ALT, alanine aminotransferase.

Model I adjust for none.

Model II adjust for age and gender.

Model III adjust for age, gender, BMI, SBP, DBP, TG, TC, HDL-C, LDL-C, BUN, Scr, smoking status, drinking status, family history of diabetes.

We divided the total population into two groups, one with normal values of liver function indicators (AST ≤ 40 U/L and ALT ≤ 40 U/L) and the other with abnormal liver function indicators (AST>40 U/L or ALT>40 U/L). Then we constructed three models to analyze the independent effects of the AST/ALT ratio on the incidence of diabetes (univariate and multivariate Cox proportional hazard model). The effect sizes (HR and 95% CI) were listed in Supplementary Table S1.

As is well known, the normal range of AST/ALT ratio is 0.8–1.5. According to this, we divided the total population into three groups. Then we constructed three models to analyze the independent effects of the AST/ALT ratio on the incidence of diabetes (univariate and multivariate Cox proportional hazard model). The effect sizes (HR and 95% CI) were listed in Supplementary Table S2.

In addition, the Kaplan–Meier curve of the cumulative hazards of incident diabetes risk stratified by AST/ALT ratio categories were presented in Supplementary Figure S1.

**Figure 3:**
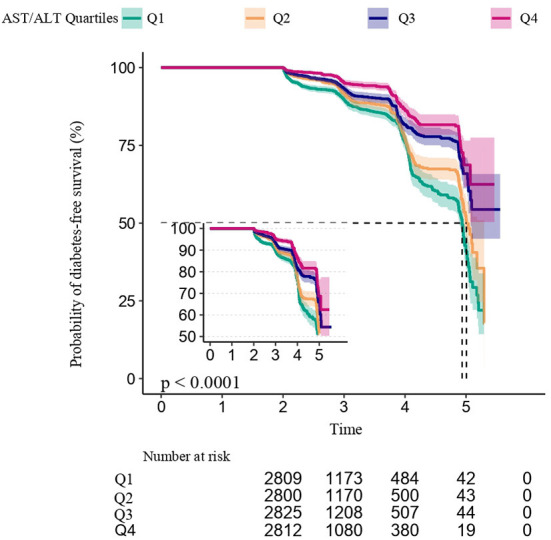
Kaplan–Meier event-free survival curve based on AST/ALT ratio quartiles and the incidence of diabetes in prediabetic patients (log-rank, *P* < 0.0001). Each color of lines indicates a quintile group. The color range indicates the 95% confidence interval (CI) range of cumulative incidence of diabetes at a different follow-up time.

Figure 3 shows the dose-response relationship between the AST/ALT ratio and the risk of diabetes in prediabetic patients. We found a decreasing trend of incidence of diabetes progressing from prediabetes with a higher AST/ALT ratio.

### Subgroup analysis

We further performed subgroup analyses to stratify the association between AST/ALT ratio and incident of diabetes by age, BMI, TG, HDL-C, and family history of diabetes as provided in Table 5. We observed that the negative relationship between the AST/ALT ratio and the risk of diabetes in the prediabetic population remained consistent across all subgroup variables. Meanwhile, we observed that only a small number of interactions including age and TG in the association between the AST/ALT ratio and the risk of diabetes in the prediabetic population (all *P*-values for interaction < 0.05). In this study, a stronger association was detected in the population with age < 45 years, and TG ≤ 1.7 mmol/L.

**Table 5:** Subgroup analysis between AST/ALT ratio and diabetes in participants with prediabetes.

**Characteristic**	**No of participants**	**Event (%)**	**HR (95%CI)**	***P*-value**	***P* for interaction**
**Age (years)**					
< 45	4,359	255 (5.8)	0.37 (0.25–0.56)	< 0.001	0.004
45–65	5,032	741 (14.7)	0.43 (0.34–0.54)	< 0.001	
>65	1,855	277 (14.9)	0.56 (0.41–0.77)	< 0.001	
**BMI (Kg/m2)**					
≤ 25	5,982	463 (7.7)	0.31 (0.24–0.41)	< 0.001	0.588
>25	5,264	810 (15.4)	0.44 (0.35–0.55)	< 0.001	
**TG (mmol/L)**					
≤ 1.70	6,973	608 (8.7)	0.30 (0.23–0.38)	< 0.001	0.002
>1.70	4,273	665 (15.6)	0.60 (0.47–0.77)	< 0.001	
**HDL-C (mmol/L)**					
≤ 1.04	1,531	201 (13.1)	0.32 (0.19–0.54)	< 0.001	0.181
>1.04	9,715	1,072 (11)	0.42 (0.35–0.50)	< 0.001	
**Family history of diabetes**					
No	10,978	1,226 (11.2)	0.40 (0.34–0.48)	< 0.001	0.865
Yes	268	47 (17.5)	0.54 (0.21–1.43)	0.215	

Results are shown as hazard ratios (HRs) with 95% confidence intervals (CIs).

BMI, body mass index; TG, triglyceride; HDL-C, high-density lipid cholesterol.

Models are adjusted for age, gender, BMI, SBP, DBP, TG, TC, HDL-C, LDL-C, BUN, Scr, smoking status, drinking status, and family history of diabetes except for the subgroup variable itself.

## Discussion

In this retrospective cohort study, we established a negative association between the AST/ALT ratio and the risk of diabetes progressing from prediabetes. These results remained stable after adjustment for all potential confounding factors. Furthermore, the negative association between the AST/ALT ratio and the risk of diabetes in prediabetic patients was more evident in participants with age < 45 years, and TG ≤ 1.7 mmol/L.

Several previous studies reported the relationship between the AST/ALT ratio and diabetes risk (29, 37, 38). A recent study investigated the relationship between AST/ALT ratio and incident T2DM in populations with or without obesity and demonstrated that non-obese individuals with AST/ALT ≤ 0.875 have a higher risk of developing T2DM than obese individuals with AST/ALT ≥ 0.875 (38). Chen et al. performed a retrospective cohort study involving 15,291 Japanese individuals from 2004 to 2015 and demonstrated that the AST/ALT ratio was negatively correlated with T2DM (HR = 0.617, 95% CI: 0.405–0.938) (29). However, inconsistent with our findings, their relationship was non-linear and had a saturation effect, and the inflection point was 0.882. We postulated that these differences were due to: i. Key differences between populations in both studies in terms of age range and ethnicity/race; ii. Variations in covariates were included as potential confounders in the studies. Similarly, a cross-sectional study of the fifth Korean National Health and Nutrition Examination Survey (KNHANES V), 2011–2016, found that the AST/ALT ratio was inversely associated with T2DM risk (39). This study found that prediabetic patients with age < 45 years old and TG ≤ 1.70 mmol/L had a lower risk of progressing into diabetes than others. Currently, we do not have an obvious explanation for this discrepancy. Possible explanations are as follows. Age growth has been shown to be an important risk factor for diabetes. Based on this, youth itself was previously considered a relative protective factor in the development of diabetes. Meanwhile, it is well known that people who tend to maintain better health are at lower risk of diabetes (40, 41). Furthermore, Abnormal lipid metabolism is associated with the pathogenesis of diabetes, which may be associated with impaired insulin reactivity and abnormal blood glucose control (42). Cui et.al found that elevated TG is an independent risk factor for T2DM incidence in the general Chinese population (43). A cross-sectional survey of 15,928 diabetic patients found that high TG patients accounted for 49.7% of the total participants (44). However, since its exact mechanism is unclear, the results of the subgroup and the interaction analysis should be interpreted with caution. And additional large trials are needed for definitive conclusions.

Biological mechanisms involved in the association between AST/ALT ratio and diabetes have not been elucidated, however, there are some potential explanations. As is well known, there have been several studies on the relationship between AST/ALT ratio and non-alcoholic fatty liver disease (NAFLD). The liver plays a crucial role in the control of gluconeogenesis, glycogenolysis, glycolysis, and gluconeogenesis, which are key steps in maintaining glucose homeostasis (45). Any damage to the liver may lead to changes in cell membrane permeability, resulting in leakage of hepatic AST and ALT into the circulatory system (46, 47). The AST/ALT ratio reflects the severity of hepatic steatosis and inflammation. Excess accumulation of fat and inflammation in the liver without heavy alcohol intake leads to NAFLD (48, 49). A recent longitudinal cohort study involving 12,127 Chinese non-obese participants reported that a lower AST/ALT ratio was independently associated with new-onset NAFLD during a 5-year follow-up (50). Moreover, ectopic deposition of lipids in hepatocytes during NAFLD (steatosis) directly or indirectly inhibits key parts of the insulin signaling pathway and significantly increases the risk of T2DM. Accumulating evidence has revealed that NAFLD is closely associated with IR and the AST/ALT ratio is indicative of insulin resistance (IR) (51–53). In a cross-sectional study involving 2,747 adults from the National Health and Nutrition Examination Survey (NHANES) 2011–2016, Visaria et al. discovered that a low AST/ALT ratio is associated with increased IR among those without liver dysfunctions (54). Comparable findings were found in the Chinese population (55). Therefore, the mechanisms involved in the correlation between AST/ALT ratio and diabetes have not been conclusively determined, which warrants further investigation.

This study has substantial strengths. First, our sample size is relatively large and is more representative of the Chinese population. Second, the follow-up duration in this cohort study was up to 6 years, which made the results more convincing. Third, we adjusted for potential confounders to minimize residual confounders in the multivariate analysis, which made the results more reliable. Fourth, sensitivity analysis was performed by handling the AST/ALT ratio as both continuous variables and categorical variables, which reduced contingency in data analysis and enhanced the stability of results. Furthermore, a subgroup analysis was conducted to ensure the robustness of the presented results. Finally, our findings have potential significant clinical implications. AST/ALT ratio is a simple, inexpensive, and routine clinical measurement, which predates traditional predictors of T2DM. It is well known that a high AST/ALT ratio may be a sign of abnormal liver function. However, a low AST/ALT ratio may not necessarily be advantageous. Our study found that a low AST/ALT ratio may increase the risk of developing diabetes. The normal range for AST/ALT ratio is 0.8–1.5. When the AST/ALT ratio is below the normal value, screening for diabetes is suggested. The findings of this study will help to identify patients at high risk of diabetes at an early stage and to make timely lifestyle modifications and interventions related to early diabetes, which may reduce the incidence of diabetes in the long term.

However, this study is associated with a few limitations. First, we only studied Chinese adults. Therefore, our conclusions may not be generalizable to other age- and ethnic groups. Second, due to the nature of the secondary analysis of published data, some important variables were not included, such as physical activities and pre-existing cardiovascular diseases. Studies should assess the relationship between AST/ALT ratio and T2DM. Third, in this study, diabetes was defined by fasting glucose levels ≥7.00 mmol/L and/or self-reported diabetes during follow-up, rather than the test of oral glucose tolerance or measurement of glycosylated hemoglobin, which may underestimate T2DM incidences. However, practically, it is not feasible to perform oral glucose tolerance tests on all participants. Fourth, this study did not classify diabetes as type 1 diabetes and T2DM. However, because T2DM accounts for about 95% of all diabetes cases, our findings are likely more representative of T2DM. Finally. the AST/ALT ratio is dynamic and changes over time. However, we only measured AST/ALT ratio at baseline.

## Conclusion

In summary, this present study suggests that a lower AST/ALT ratio is independently associated with a higher risk of diabetes onset in Chinese adults with prediabetes. Regular monitoring of AST/ALT ratio dynamics can help avoid progression to diabetes from prediabetics. The AST/ALT ratio might thus be a useful tool for detecting prediabetic individuals at a high risk of developing diabetes. However, further prospective studies are needed to validate our study findings.

## Data availability statement

The original contributions presented in the study are included in the article/Supplementary material, further inquiries can be directed to the corresponding authors.

## Ethics statement

The studies involving human participants were reviewed and approved by the Rich Healthcare Group Review Board. The patients/participants provided their written informed consent to participate in this study. Written informed consent was obtained from the individual(s) for the publication of any potentially identifiable images or data included in this article.

## Author contributions

JC and XW conceived the study idea. HL and LJ analyzed the data. HZ and HL reviewed the literature and wrote the first draft. LJ and HZ critically reviewed, edited the manuscript, and approved the final version. All authors read and approved the final manuscript.

## Supplementary material

Supplementary Table1 Association between AST/ALT ratio and the risk of diabetes progressing from prediabetes by value of liver function.

Supplementary Table2 Relationship between AST/ALT ratio and the risk of diabetes in prediabetic patients in different models.

Supplementary Figure S1 Kaplan–Meier curve of the cumulative hazards of incident diabetes risk stratified by AST/ALT ratio categories in patients with prediabetes (log-rank, *P* < 0.0001). Each color of lines indicates a quintile group. The color range indicates the 95% confidence interval (CI) range of cumulative incidence of diabetes at a different follow-up time.
