# Supplementary figures and images for: Association between aspartate aminotransferase to alanine aminotransferase ratio and the risk of diabetes in Chinese prediabetic population: A retrospective cohort study
Source: Front Public Health. 2023 Jan 4;10:1045141. doi: 10.3389/fpubh.2022.1045141 (PMC9846751; doi:10.3389/fpubh.2022.1045141)

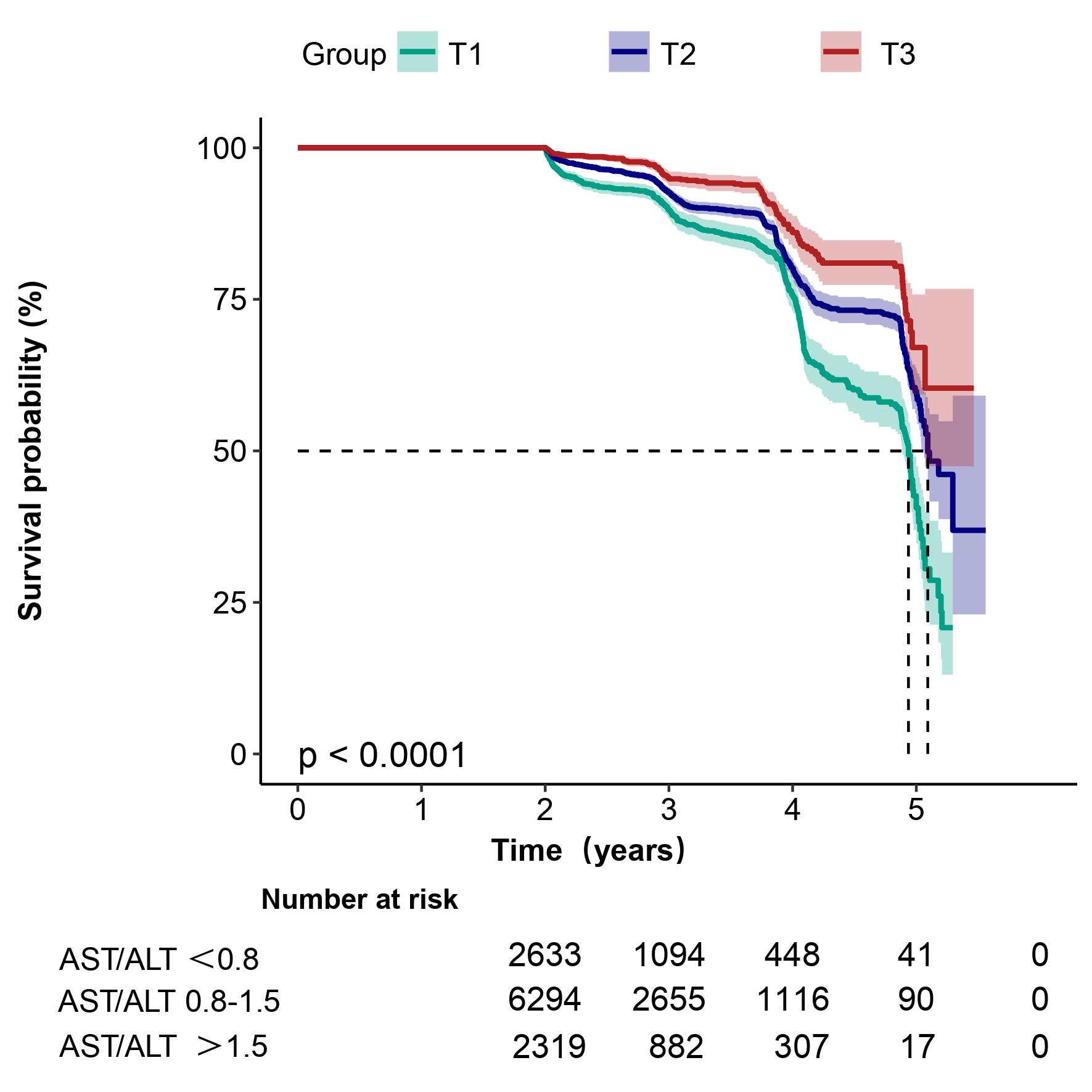

Supplement: Supplementary Figure S1 — Kaplan–Meier curve of the cumulative hazards of incident diabetes risk stratified by AST/ALT ratio categories in patients with prediabetes (log-rank, P < 0.0001). Each color of lines indicates a quintile group. The color range indicates the 95% confidence interval (CI) range of cumulative incidence of diabetes at a different follow-up time. [file Image_1.jpeg]
