# Supplementary material for: Association between aspartate aminotransferase to alanine aminotransferase ratio and the risk of diabetes in Chinese prediabetic population: A retrospective cohort study
Source: Front Public Health. 2023 Jan 4;10:1045141. doi: 10.3389/fpubh.2022.1045141 (PMC9846751; doi:10.3389/fpubh.2022.1045141)
Supplement: Supplementary file 5 [file Table_4.DOCX]

Multiple regression equations

| Exposure | Non-adjusted | Adjust I | Adjust II |
| --- | --- | --- | --- |
| AST.ALT | 0.49 (0.42, 0.56) <0.0001 | 0.31 (0.26, 0.37) <0.0001 | 0.40 (0.33, 0.47) <0.0001 |

Data：HR (95% CI) Pvalue *P<0.05 **P<0.01 ***P<0.001

Outcome: CENSOR.OF.DIABETES.AT.FOLLOWUP.1.YES.0.NO

Exposure: AST.ALT

Non-adjusted model adjust for: None

Adjust I model adjust for: AGE.Y; GENDER.1.MALE.2.FEMALE

Adjust II model adjust for: AGE.Y;BMI.KG.M2;GENDER.1.MALE.2.FEMALE;TRIGLYCERIDE.MMOL.L;HDL.C.MMOL.L;SBP.MMHG;DBP.MMHG;CHOLESTEROL.MMOL.L;BUN.MMOL.L;CCR.UMOL.L;SMOKING.STATUS.1.CURRENT.SMOKER.2.EVER.SMOKER.3.NEVER.SMOKER;DRINKING.STATUS.1.CURRENT.DRINKER.2.EVER.DRINKER.3.NEVER.DRINKER;FAMILY.HISTROY.OF.DIABETES.1.YES.0.NO

Cox model time: YEAR.OF.FOLLOWUP

| Outcome | Exposure | Non-adjusted | Adjust I | Adjust II |
| --- | --- | --- | --- | --- |
| CENSOR.OF.DIABETES.AT.FOLLOWUP.1.YES.0.NO | AST.ALT | 11246 | 11246 | 11246 |

Multiple regression equations

| Exposure | Non-adjusted | Adjust I | Adjust II |
| --- | --- | --- | --- |
| AST.ALT group |  |  |  |
| 0.285562633 - 0.815489749 | 1.0 | 1.0 | 1.0 |
| 0.815789474 - 1.082926829 | 0.80 (0.69, 0.92) 0.0013 | 0.63 (0.54, 0.72) <0.0001 | 0.71 (0.62, 0.82) <0.0001 |
| 1.083333333 - 1.414814815 | 0.59 (0.51, 0.68) <0.0001 | 0.41 (0.35, 0.49) <0.0001 | 0.50 (0.42, 0.59) <0.0001 |
| 1.414893617 - 9.777777778 | 0.43 (0.36, 0.51) <0.0001 | 0.27 (0.22, 0.33) <0.0001 | 0.35 (0.29, 0.43) <0.0001 |
| AST.ALT group trend | 0.42 (0.36, 0.50) <0.0001 | 0.27 (0.22, 0.32) <0.0001 | 0.35 (0.29, 0.42) <0.0001 |

DATA：HR (95% CI) Pvalue *P<0.05 **P<0.01 ***P<0.001

OUTCOME： CENSOR.OF.DIABETES.AT.FOLLOWUP.1.YES.0.NO

Exposure: AST.ALT group; AST.ALT group trend

Non-adjusted model adjust for: None

Adjust I model adjust for: AGE.Y; GENDER.1.MALE.2.FEMALE

Adjust II model adjust for: AGE.Y;BMI.KG.M2;GENDER.1.MALE.2.FEMALE;TRIGLYCERIDE.MMOL.L;HDL.C.MMOL.L;SBP.MMHG;DBP.MMHG;CHOLESTEROL.MMOL.L;BUN.MMOL.L;CCR.UMOL.L;SMOKING.STATUS.1.CURRENT.SMOKER.2.EVER.SMOKER.3.NEVER.SMOKER;DRINKING.STATUS.1.CURRENT.DRINKER.2.EVER.DRINKER.3.NEVER.DRINKER;FAMILY.HISTROY.OF.DIABETES.1.YES.0.NO

Cox model time: YEAR.OF.FOLLOWUP

| Outcome | Exposure | Non-adjusted | Adjust I | Adjust II |
| --- | --- | --- | --- | --- |
| CENSOR.OF.DIABETES.AT.FOLLOWUP.1.YES.0.NO | AST.ALT group | 11246 | 11246 | 11246 |
| CENSOR.OF.DIABETES.AT.FOLLOWUP.1.YES.0.NO | AST.ALT group trend | 11246 | 11246 | 11246 |

Description of study population

| AST.ALT quartile | Q1 | Q2 | Q3 | Q4 | P-value | P-value* |
| --- | --- | --- | --- | --- | --- | --- |
| N | 2809 | 2800 | 2825 | 2812 |  |  |
| AGE.Y | 44.28 ± 11.16 | 49.85 ± 12.90 | 52.28 ± 14.22 | 53.36 ± 15.64 | <0.001 | <0.001 |
| BMI.KG.M2 | 26.49 ± 3.15 | 25.31 ± 3.12 | 24.31 ± 3.10 | 23.20 ± 3.08 | <0.001 | <0.001 |
| SBP.MMHG | 128.41 ± 16.00 | 127.57 ± 17.28 | 127.80 ± 18.52 | 126.62 ± 18.92 | 0.002 | <0.001 |
| DBP.MMHG | 80.36 ± 10.70 | 78.89 ± 10.89 | 78.05 ± 11.31 | 76.41 ± 11.40 | <0.001 | <0.001 |
| CHOLESTEROL.MMOL.L | 5.09 ± 0.96 | 4.98 ± 0.93 | 4.96 ± 0.94 | 4.92 ± 0.95 | <0.001 | <0.001 |
| TRIGLYCERIDE.MMOL.L | 1.82(1.22,2.72) | 1.62(1.14,2.34) | 1.33(0.92,1.94) | 1.14(0.82,1.48) | <0.001 | <0.001 |
| ALT.U.L | 50.98 ± 31.14 | 27.28 ± 11.19 | 19.75 ± 7.35 | 13.80 ± 5.89 | <0.001 | <0.001 |
| AST.U.L | 31.34 ± 14.83 | 25.58 ± 9.94 | 24.22 ± 8.56 | 24.16 ± 10.47 | <0.001 | <0.001 |
| AST.ALT | 0.65 ± 0.11 | 0.95 ± 0.08 | 1.24 ± 0.10 | 1.81 ± 0.47 | <0.001 | <0.001 |
| BUN.MMOL.L | 5.06 ± 1.19 | 5.09 ± 1.23 | 5.02 ± 1.29 | 4.99 ± 1.34 | 0.013 | <0.001 |
| CCR.UMOL.L | 77.03 ± 14.02 | 75.31 ± 15.74 | 73.14 ± 16.23 | 70.67 ± 17.53 | <0.001 | <0.001 |
| YEAR.OF.FOLLOWUP | 2.96 ± 0.92 | 3.00 ± 0.92 | 3.04 ± 0.95 | 2.96 ± 0.87 | <0.001 | 0.008 |
| GENDER.1.MALE.2.FEMALE |  |  |  |  | <0.001 | - |
| 1 | 2431 (86.54%) | 2075 (74.11%) | 1719 (60.85%) | 1291 (45.91%) |  |  |
| 2 | 378 (13.46%) | 725 (25.89%) | 1106 (39.15%) | 1521 (54.09%) |  |  |
| SMOKING.STATUS.1.CURRENT.SMOKER.2.EVER.SMOKER.3.NEVER.SMOKER |  |  |  |  | <0.001 | - |
| 1 | 806 (28.69%) | 657 (23.46%) | 531 (18.80%) | 386 (13.73%) |  |  |
| 2 | 176 (6.27%) | 131 (4.68%) | 108 (3.82%) | 74 (2.63%) |  |  |
| 3 | 1827 (65.04%) | 2012 (71.86%) | 2186 (77.38%) | 2352 (83.64%) |  |  |
| DRINKING.STATUS.1.CURRENT.DRINKER.2.EVER.DRINKER.3.NEVER.DRINKER |  |  |  |  | <0.001 | - |
| 1 | 116 (4.13%) | 117 (4.18%) | 129 (4.57%) | 137 (4.87%) |  |  |
| 2 | 701 (24.96%) | 572 (20.43%) | 476 (16.85%) | 313 (11.13%) |  |  |
| 3 | 1992 (70.91%) | 2111 (75.39%) | 2220 (78.58%) | 2362 (84.00%) |  |  |
| FAMILY.HISTROY.OF.DIABETES.1.YES.0.NO |  |  |  |  | 0.007 | - |
| 0 | 2720 (96.83%) | 2735 (97.68%) | 2775 (98.23%) | 2748 (97.72%) |  |  |
| 1 | 89 (3.17%) | 65 (2.32%) | 50 (1.77%) | 64 (2.28%) |  |  |
| CENSOR.OF.DIABETES.AT.FOLLOWUP.1.YES.0.NO |  |  |  |  | <0.001 | - |
| 0 | 2366 (84.23%) | 2434 (86.93%) | 2537 (89.81%) | 2636 (93.74%) |  |  |
| 1 | 443 (15.77%) | 366 (13.07%) | 288 (10.19%) | 176 (6.26%) |  |  |

## Univariate analysis

|  | Statistics | CENSOR.OF.DIABETES.AT.FOLLOWUP.1.YES.0.NO |
| --- | --- | --- |
| AGE.Y | 49.94 ± 14.03 | 1.03 (1.02, 1.03) <0.0001 |
| GENDER.1.MALE.2.FEMALE |  |  |
| 1 | 7516 (66.83%) | 1.0 |
| 2 | 3730 (33.17%) | 0.89 (0.79, 1.01) 0.0632 |
| BMI.KG.M2 | 24.82 ± 3.34 | 1.11 (1.09, 1.12) <0.0001 |
| SBP.MMHG | 127.60 ± 17.73 | 1.02 (1.01, 1.02) <0.0001 |
| DBP.MMHG | 78.43 ± 11.17 | 1.02 (1.01, 1.02) <0.0001 |
| CHOLESTEROL.MMOL.L | 4.99 ± 0.95 | 1.04 (0.98, 1.10) 0.2145 |
| TRIGLYCERIDE.MMOL.L | 1.77 ± 1.40 | 1.12 (1.09, 1.15) <0.0001 |
| HDL.C.MMOL.L | 1.34 ± 0.32 | 1.55 (1.39, 1.73) <0.0001 |
| LDL.MMOL.L | 2.89 ± 0.70 | 1.00 (0.93, 1.08) 0.9327 |
| ALT.U.L | 27.94 ± 22.25 | 1.01 (1.01, 1.01) <0.0001 |
| AST.ALT | 1.16 ± 0.50 | 0.49 (0.42, 0.56) <0.0001 |
| BUN.MMOL.L | 5.04 ± 1.26 | 1.04 (0.99, 1.08) 0.1058 |
| CCR.UMOL.L | 74.04 ± 16.10 | 1.00 (1.00, 1.01) 0.2942 |
| SMOKING.STATUS.1.CURRENT.SMOKER.2.EVER.SMOKER.3.NEVER.SMOKER |  |  |
| 1 | 2380 (21.16%) | 1.0 |
| 2 | 489 (4.35%) | 1.34 (1.06, 1.70) 0.0141 |
| 3 | 8377 (74.49%) | 0.81 (0.71, 0.92) 0.0008 |
| DRINKING.STATUS.1.CURRENT.DRINKER.2.EVER.DRINKER.3.NEVER.DRINKER |  |  |
| 1 | 499 (4.44%) | 1.0 |
| 2 | 2062 (18.34%) | 0.65 (0.51, 0.83) 0.0005 |
| 3 | 8685 (77.23%) | 0.67 (0.54, 0.84) 0.0004 |
| FAMILY.HISTROY.OF.DIABETES.1.YES.0.NO |  |  |
| 0 | 10978 (97.62%) | 1.0 |
| 1 | 268 (2.38%) | 1.59 (1.19, 2.12) 0.0019 |
| YEAR.OF.FOLLOWUP | 2.99 ± 0.92 | 0.00 (0.00, 0.00) <0.0001 |

Data：HR (95% CI) Pvalue *P<0.05 **P<0.01 ***P<0.001

outcome: CENSOR.OF.DIABETES.AT.FOLLOWUP.1.YES.0.NO

exposure: AGE.Y; GENDER.1.MALE.2.FEMALE; BMI.KG.M2; SBP.MMHG; DBP.MMHG; CHOLESTEROL.MMOL.L; TRIGLYCERIDE.MMOL.L; HDL.C.MMOL.L; LDL.MMOL.L; ALT.U.L; AST.ALT; BUN.MMOL.L; CCR.UMOL.L; SMOKING.STATUS.1.CURRENT.SMOKER.2.EVER.SMOKER.3.NEVER.SMOKER; DRINKING.STATUS.1.CURRENT.DRINKER.2.EVER.DRINKER.3.NEVER.DRINKER; FAMILY.HISTROY.OF.DIABETES.1.YES.0.NO; YEAR.OF.FOLLOWUP

Adjustment variable: None

Cox model time: YEAR.OF.FOLLOWUP

| Exposure | CENSOR.OF.DIABETES.AT.FOLLOWUP.1.YES.0.NO |
| --- | --- |
| AGE.Y | 11246 |
| GENDER.1.MALE.2.FEMALE | 11246 |
| BMI.KG.M2 | 11246 |
| SBP.MMHG | 11246 |
| DBP.MMHG | 11246 |
| CHOLESTEROL.MMOL.L | 11246 |
| TRIGLYCERIDE.MMOL.L | 11246 |
| HDL.C.MMOL.L | 11246 |
| LDL.MMOL.L | 11246 |
| ALT.U.L | 11246 |
| AST.ALT | 11246 |
| BUN.MMOL.L | 11246 |
| CCR.UMOL.L | 11246 |
| SMOKING.STATUS.1.CURRENT.SMOKER.2.EVER.SMOKER.3.NEVER.SMOKER | 11246 |
| DRINKING.STATUS.1.CURRENT.DRINKER.2.EVER.DRINKER.3.NEVER.DRINKER | 11246 |
| FAMILY.HISTROY.OF.DIABETES.1.YES.0.NO | 11246 |
| YEAR.OF.FOLLOWUP | 11246 |

Description of study population

| CENSOR.OF.DIABETES.AT.FOLLOWUP.1.YES.0.NO | 0 | 1 | Standardize diff. | P-value | P-value* |
| --- | --- | --- | --- | --- | --- |
| N | 9973 | 1273 |  |  |  |
| AGE.Y | 49.23 ± 14.07 | 55.58 ± 12.35 | 0.48 (0.42, 0.54) | <0.001 | <0.001 |
| BMI.KG.M2 | 24.65 ± 3.31 | 26.16 ± 3.31 | 0.45 (0.40, 0.51) | <0.001 | <0.001 |
| SBP.MMHG | 126.95 ± 17.51 | 132.73 ± 18.58 | 0.32 (0.26, 0.38) | <0.001 | <0.001 |
| DBP.MMHG | 78.10 ± 11.07 | 80.98 ± 11.59 | 0.25 (0.20, 0.31) | <0.001 | <0.001 |
| CHOLESTEROL.MMOL.L | 4.98 ± 0.94 | 5.07 ± 0.97 | 0.10 (0.04, 0.16) | <0.001 | 0.002 |
| TRIGLYCERIDE.MMOL.L | 1.40 (0.91,2.14) | 1.72(1.21,2.58) | 0.29 (0.23, 0.35) | <0.001 | <0.001 |
| ALT.U.L | 27.00 ± 21.12 | 35.31 ± 28.59 | 0.33 (0.27, 0.39) | <0.001 | <0.001 |
| AST.U.L | 25.95 ± 11.18 | 29.26 ± 14.00 | 0.26 (0.20, 0.32) | <0.001 | <0.001 |
| AST.ALT | 1.18 ± 0.50 | 1.02 ± 0.43 | 0.34 (0.28, 0.40) | <0.001 | <0.001 |
| BUN.MMOL.L | 5.04 ± 1.26 | 5.07 ± 1.30 | 0.03 (-0.03, 0.09) | 0.328 | 0.490 |
| CCR.UMOL.L | 74.03 ± 15.96 | 74.11 ± 17.17 | 0.01 (-0.05, 0.06) | 0.861 | 0.696 |
| YEAR.OF.FOLLOWUP | 2.95 ± 0.91 | 3.29 ± 0.93 | 0.37 (0.31, 0.42) | <0.001 | <0.001 |
| GENDER.1.MALE.2.FEMALE |  |  | 0.11 (0.05, 0.17) | <0.001 | - |
| 1 | 6608 (66.26%) | 908 (71.33%) |  |  |  |
| 2 | 3365 (33.74%) | 365 (28.67%) |  |  |  |
| SMOKING.STATUS.1.CURRENT.SMOKER.2.EVER.SMOKER.3.NEVER.SMOKER |  |  | 0.23 (0.17, 0.29) | <0.001 | - |
| 1 | 2027 (20.32%) | 353 (27.73%) |  |  |  |
| 2 | 402 (4.03%) | 87 (6.83%) |  |  |  |
| 3 | 7544 (75.64%) | 833 (65.44%) |  |  |  |
| DRINKING.STATUS.1.CURRENT.DRINKER.2.EVER.DRINKER.3.NEVER.DRINKER |  |  | 0.13 (0.07, 0.19) | <0.001 | - |
| 1 | 412 (4.13%) | 87 (6.83%) |  |  |  |
| 2 | 1811 (18.16%) | 251 (19.72%) |  |  |  |
| 3 | 7750 (77.71%) | 935 (73.45%) |  |  |  |
| FAMILY.HISTROY.OF.DIABETES.1.YES.0.NO |  |  | 0.09 (0.03, 0.15) | 0.001 | - |
| 0 | 9752 (97.78%) | 1226 (96.31%) |  |  |  |
| 1 | 221 (2.22%) | 47 (3.69%) |  |  |  |
